# Curcumin protects high glucose-induced diabetic myocardial injury by regulating Nrf2 and PI3K/AKT pathways

**DOI:** 10.3389/fphar.2026.1752397

**Published:** 2026-06-12

**Authors:** ZeYue Xu, ZhuangYin Qu, Xia Wu

**Affiliations:** 1 Department of Pharmacy, Nanjing First Hospital, Nanjing Medical University, Nanjing, China; 2 School of Basic Medicine and Clinical Pharmacy, China Pharmaceutical University, Nanjing, China

**Keywords:** curcumin, diabetic cardiomyopathy, myocardial protection, Nrf2, PI3K/AKT

## Abstract

Hyperglycemia can lead to excessive production of reactive oxygen species (ROS), contributing to diabetes and its complications, such as diabetic cardiomyopathy (DCM). This study aims to elucidate the protective mechanisms of Curcumin (CUR) against hyperglycemia-induced cardiomyocyte injury. We constructed a high-glucose (HG) model using primary cardiomyocytes and determined the optimal concentration of CUR by assessing cell viability with the CCK-8 assay. Enzyme-linked assays were used to measure the activities of HO-1, T-SOD, and GSH-Px, investigating the antioxidant effects of the Nrf2 signaling pathway. Flow cytometry was employed to measure apoptosis and mitochondrial membrane potential (ΔΨm). Mitochondria were isolated from cardiomyocytes to examine cytosolic and mitochondrial cytochrome c (cytc) expression. Western blotting was used to analyze the expression levels of Nrf2, PI3K, AKT, Keap1, HO-1, Bcl-2, and Bax proteins. Our findings suggest that CUR enhanced the viability and antioxidant enzyme activity of primary cardiomyocytes under HG conditions, stabilized ΔΨm, reduced ROS production, and decreased apoptosis. Moreover, CUR alleviated HG-induced cardiomyocyte damage by upregulating Nrf2, PI3K, AKT, HO-1, and Bcl-2 expression, while downregulating Bax. These findings demonstrate that the protective effect of CUR against diabetic cardiac injury may be mediated through activation of the Nrf2 and PI3K/AKT signaling pathways and upregulation of antioxidant enzyme expression, aiming to provide a theoretical foundation for further research into the therapeutic application of CUR in DCM.

## 1 Introduction

Diabetes mellitus poses a major global health challenge and is associated with multiple severe complications due to chronic hyperglycemia, including diabetic cardiomyopathy (DCM), a leading cause of heart failure in diabetic patients (Jia et al., 2018). DCM is characterized by myocardial structural and functional abnormalities that occur independently of hypertension and coronary artery disease (Deng et al., 2025). A primary driver of these abnormalities is high-glucose (HG)-induced oxidative stress, which stimulates excessive reactive oxygen species (ROS) production. This oxidative burden directly damages cellular structures, disrupts metabolic pathways, and triggers apoptosis, all contributing to the progression of DCM (Rampin et al., 2022).

Recent studies highlight the importance of the Nrf2 (nuclear factor erythroid 2-related factor 2) signaling pathway in cellular defense against oxidative stress. Nrf2 is a transcription factor that regulates the expression of various antioxidant enzymes, including heme oxygenase-1 (HO-1), superoxide dismutase (SOD), and glutathione peroxidase (GSH-Px), to mitigate oxidative injury (Fiori et al., 2025). Upon activation by oxidative stress, Nrf2 translocates to the nucleus, where it binds to antioxidant response elements (AREs) within gene promoters, initiating the transcription of key antioxidant proteins (Patel et al., 2020). Given its central role, Nrf2 activation is increasingly recognized as a promising therapeutic target for protecting the myocardium from hyperglycemia-induced damage in diabetic patients (Wu et al., 2022).

Curcumin (CUR), a natural polyphenol isolated from *Curcuma longa*, is well known for its potent antioxidant and anti-inflammatory properties (Wu et al., 2025). Extensive research indicates that CUR exerts protective effects in various cell types by regulating oxidative stress pathways, including Nrf2 (He et al., 2012). In cardiac cells, CUR has been shown to alleviate oxidative damage, reduce apoptosis, and stabilize cellular functions, underscoring its potential as a cardioprotective agent in diabetic conditions (Rahban et al., 2020).

In this study, we explored the effects of CUR on primary cardiomyocytes under HG conditions, modeling the cellular stress environment observed in DCM. Specifically, we aimed to determine the optimal concentration of CUR for cellular protection, evaluate its impact on antioxidant enzyme activity, and assess its influence on cell viability, mitochondrial membrane potential (ΔΨm), ROS production, and apoptosis. This study aims to determine whether CUR confers cardioprotection in primary cardiomyocytes under diabetic conditions by simultaneously activating Nrf2 and PI3K/AKT signaling, and to elucidate if these pathways function independently or via a synergistic crosstalk mechanism. These findings could contribute to the development of novel therapeutic strategies for managing DCM and improving cardiovascular outcomes in diabetic patients.

## 2 Materials and methods

### 2.1 Materials

CUR was purchased from Aladdin (Shanghai, China), it was dissolved in dimethyl sulfoxide (DMSO) to prepare a stock solution. The final concentration of DMSO in the culture medium was less than 0.1% (v/v). LV-Nrf2-shRNA was purchased from Jinkairui Biological Company (Wuhan, China). Nrf2 and Keap1 antibodies were purchased from Abcam (Abcam, CA, USA). The β-actin antibody was purchased from Santa Cruz Biotechnology (Santa Cruz, CA, USA). PI3K, AKT, Keap1, HO-1, Bax, and Bcl-2 antibodies were purchased from Cell Signaling Technology (Cell Signaling, CA, USA).

### 2.2 Primary culture of neonatal rat cardiomyocytes

All experimental procedures adhered to the National Institutes of Health (NIH) guidelines and were approved by the Animal Care and Use Committee of Nanjing Medical University (Approval Number: NMUC20241068). Neonatal rat cardiomyocytes were isolated from 1-day-old Sprague-Dawley rats as previously described (Zhou et al., 2014). Cells were cultured in DMEM medium containing either 5.5 mmol/L or 33 mmol/L glucose, 10% fetal bovine serum (FBS), 1 U/mL penicillin, and 1 mg/mL streptomycin, in a humidified atmosphere with 5% CO_2_ at 37 °C. Culture was maintained for 3 days until the cells reached 90% confluence. Cardiomyocytes were collected via trypsin digestion, followed by continuous culture in fresh plates or prepared for subsequent experiments.

### 2.3 Lentiviral transfection

Lv-shRNA-Nrf2 or shRNA negative control (NC) constructs were transfected into cardiomyocytes cultured in fresh DMEM medium. Cells were incubated at 37 °C in 95% oxygen and 5% CO_2_ for 48 h, after which the medium containing virus was replaced with fresh medium. Cells were subsequently treated according to different experimental conditions.

### 2.4 CCK-8 assay

Cell viability was assessed using a CCK-8 assay kit. Cardiomyocytes were seeded in a 96-well plate and cultured in a 37 °C cell incubator. After adhesion, the normal medium was replaced with high-glucose medium, followed by the addition of various concentrations of CUR for 6, 12, 24, and 36 h, respectively. The medium was then removed, and cells were washed three times with PBS. Viable cells were quantified using the CCK-8 assay (Solarbio Science Technology) according to the manufacturer’s instructions. The optical density of each well was measured at 450 nm using a microplate reader (Bio-Rad, USA).

### 2.5 Reactive oxygen species detection

Cardiomyocytes were cultured in 60 mm dishes. After removing the cell culture medium, cells were washed once with room temperature PBS, and 1 mL of diluted DCFH-DA (KeyGEN BioTECH, China) was added. The cells were incubated at 37 °C for 20 min, and then washed three times with serum-free medium to remove excess DCFH-DA. ROS production was observed using a fluorescence microscope. Data are presented as mean fluorescence intensity (MFI) normalized to the Control group (set to 1.0). At least 10,000 events per sample were acquired.

### 2.6 Antioxidant activity detection

Cell samples were collected, and the activities of SOD, HO-1, MDA, and GSH-Px were measured at 550 nm, 450 nm, 550 nm, and 450 nm, respectively, using commercial kits (Elabscience, Wuhan, China). A microplate reader was used for the measurements.

### 2.7 Mitochondrial membrane potential (ΔΨm) detection

Primary neonatal rat cardiomyocytes in good condition were seeded in 6-well culture plates at a density of 2 × 10^5^ cells/well and cultured overnight at 37 °C with 5% CO_2_. Cells were treated according to the experimental conditions and cultured for 36 h. JC-1 staining working solution was added, and analysis was performed by flow cytometry. Mitochondrial membrane potential was expressed as the ratio of red (aggregates) to green (monomers) fluorescence intensity. The ratio in the Control group was set to 1.0, and experimental groups were normalized accordingly.

### 2.8 Apoptosis detection

Cardiomyocyte suspension was plated at 4 × 10^4^ cells/well in 6-well plates for 36 h according to experimental conditions. Cells were collected and incubated with 5 μL annexin V-FITC, followed by 5 μL propidium iodide. Apoptosis was assessed by flow cytometry. Apoptosis rate was calculated as the percentage of Annexin V-positive cells (early apoptosis + late apoptosis) relative to total cells. Quadrant gates were set based on unstained and single-stained controls.

### 2.9 Cytosolic cytochrome c (cytc) detection

Primary cardiomyocytes were treated for 36 h according to experimental conditions. Cells were collected and homogenized with 1–2.5 mL of mitochondrial separation reagent, followed by centrifugation to collect the cell suspension. Mitochondrial and cytosolic fractions were separated and analyzed by Western blot.

### 2.10 Western blotting

Total protein and mitochondrial protein were extracted using a protein extraction kit and mitochondrial isolation kit (Applygen Technologies Inc., Beijing, China). Cells were lysed at 4 °C, and protein concentration was determined using the BCA method. Protein samples were separated on 8%–10% SDS-PAGE gels, transferred to nitrocellulose membranes, and incubated with primary antibodies for Nrf2, Keap1, PI3K, AKT, HO-1, Bax, Bcl-2, and β-actin. The membrane was incubated with secondary antibodies conjugated to horseradish peroxidase. Protein band intensities were analyzed using Image Lab 4.0.1 (Bio-Rad Laboratories, Inc., Hercules, CA, USA). Relative protein levels were normalized to β-actin. For all Western blot experiments, protein bands were quantified using ImageJ software (NIH). The following normalization procedures were applied: Target protein normalization: Each target protein (e.g., Nrf2, HO-1, Bax, Bcl-2) was first normalized to a loading control (β-actin) from the same lane, expressed as the ratio of target protein to loading control. Each Western blot experiment was repeated using three independent biological replicates, and the quantified data represent the mean ± S. E. M from these three independent experiments.

### 2.11 Statistical analysis

All data were first tested for normality using the Shapiro–Wilk test. Homogeneity of variance was assessed using Levene’s test. For comparisons among multiple groups, one-way ANOVA was performed. When significant differences were detected, Tukey’s *post hoc* test was applied for pairwise comparisons. Data are presented as mean ± S. E. M. A *p*-value < 0.05 was considered statistically significant.

## 3 Results

### 3.1 Effect of CUR on cardiomyocyte proliferation induced by high glucose

**FIGURE 1:**
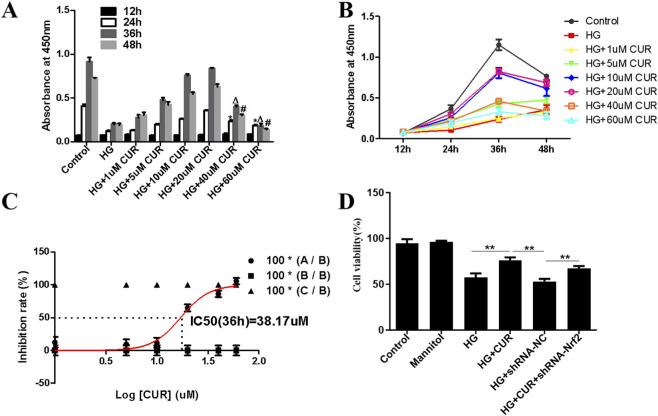
Effect of CUR on the viability and proliferation of HG-induced primary cardiomyocytes. **(A)** Different concentrations of CUR (1, 5, 10, 20, 40, 80 μM) were tested. Cells were cultured for 12, 24, 36, and 48 h, and OD values were measured using CCK-8 reagent (Note: Mannitol vs. control group showed no statistical significance, indicating that the damage was caused by HG.) **(B)** Cell growth curves showed increased OD values with culture time at CUR concentrations of 1, 5, 10, and 20 μM, while OD values decreased significantly at 40 and 80 μM. **(C)** IC50 curve for primary cardiomyocytes treated with CUR for 36 h. **(D)** Percentage of cell viability in the HG + CUR group was significantly higher than in the HG group, and in the HG + CUR + shRNA-Nrf2 group, significantly higher than in the HG + shRNA NC group (Data are presented as mean ± S. E. M; The quantitative data were obtained from all three replicate experiments., *P < 0.05, Δ*P* < 0.05, and #*P* < 0.05 vs. 20 μM CUR group at 12, 24, 36, 48 h, respectively, **P < 0.01).

The CCK-8 assay revealed that at CUR concentrations of 40 and 80 μM, the optical density (OD) values of the cells decreased significantly, with the effect becoming more pronounced over time. This reduction was statistically significant (*P* < 0.05) (Figures 1A,B). After data transformation and statistical analysis, we determined that the IC50 of CUR at 36 h was 38.17 μM (Figure 1C). In cell viability tests, Nrf2 was silenced through lentiviral transfection. Compared with the HG group, cell viability in both the HG + CUR group and the HG + CUR + shRNA-Nrf2 group was significantly increased (*P* < 0.01). These findings suggest that CUR promotes the proliferation of cardiomyocytes under high glucose (HG) conditions (Figure 1D).

### 3.2 Effects of CUR on ROS production and antioxidant enzyme activity in HG-induced cardiomyocytes

**FIGURE 2:**
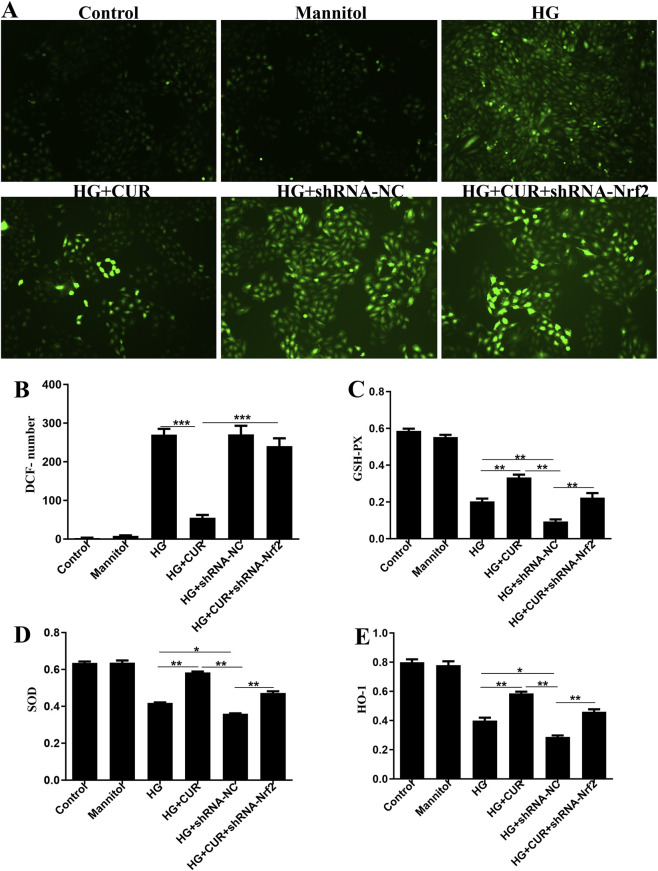
Effects of CUR on intracellular ROS production and antioxidant enzyme activity after HG-induced myocardial injury. **(A)** Fluorescent images show ROS production in myocardial cells across different intervention groups, with higher intracellular ROS levels corresponding to more intense green fluorescence. **(B)** Quantitative analysis of ROS production among different groups. **(C–E)** Quantitative analyses of HO-1, GSH-Px, and SOD levels across various intervention groups (data are presented as mean ± S. E. M; The quantitative data were obtained from all three replicate experiments. *P < 0.05, **P < 0.01, ***P < 0.001).

Previous studies have demonstrated that HG exposure increases ROS levels, which can lead to apoptosis (Zhou et al., 2019). In this study, we simulated HG conditions in primary cardiomyocyte cultures and assessed ROS production following CUR treatment. Under microscopic examination, ROS expression was indicated by green fluorescence, with minimal fluorescence observed in cells with normal morphology, suggesting low ROS levels (Figure 2A). Statistical analysis showed that ROS levels in the HG + CUR group were significantly lower than those in the HG group. ROS levels in the HG + shRNA NC group were comparable to those in the HG group, with no significant difference. This indicates that the shRNA vector alone did not affect ROS production. In the HG + CUR + shRNA-Nrf2 group, ROS levels were similar to those in the HG group but were significantly higher than those in the HG + CUR group (*P* < 0.05) (Figure 2B). These results suggest that Nrf2 knockdown abrogated the inhibitory effect of CUR on ROS generation. Antioxidant enzyme assays revealed that the activities of GSH-Px, SOD, and HO-1 in the HG + CUR group were significantly higher than those in the HG group. Similar increases in enzyme activities were observed in the HG + CUR + shRNA-Nrf2 group compared to the HG + shRNA NC group, also in the HG + CUR group compared to the HG + shRNA NC group (*P* < 0.05) (Figures 2C–E).

### 3.3 Effect of CUR on mitochondrial membrane potential (ΔΨm) in HG-induced cardiomyocytes

**FIGURE 3:**
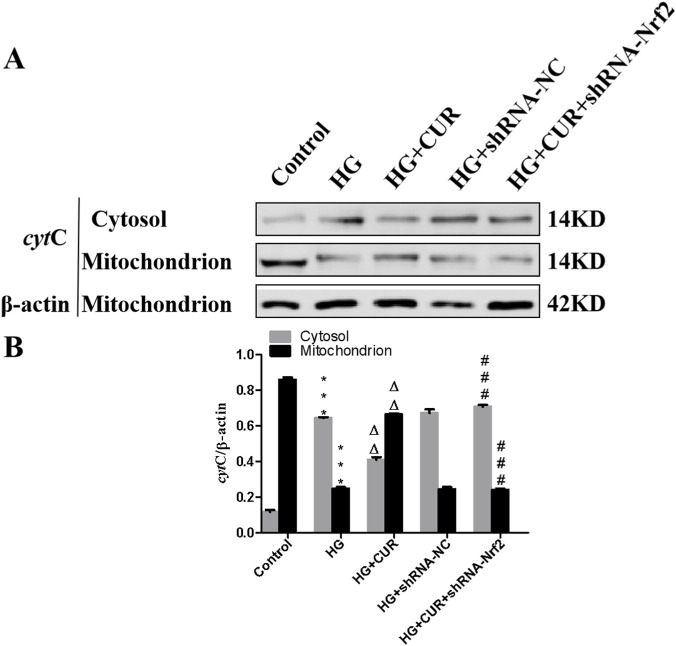
Changes in mitochondrial membrane potential of primary neonatal rat cardiomyocytes induced by HG, detected by JC-1 probe. **(A)** Images showing mitochondrial membrane potential changes across intervention groups. **(B)** Quantitative analysis of mitochondrial membrane potential among different groups (data are presented as mean ± S. E. M; The quantitative data were obtained from all three replicate experiments. *P < 0.05).

We used the JC-1 probe to assess changes in mitochondrial membrane potential (ΔΨm) in cardiomyocytes under HG conditions. The fluorescence ratio (upper right quadrant to lower right quadrant) was used to evaluate ΔΨm. Compared with the control group, ΔΨm was significantly reduced in the HG group and the HG + CUR + shRNA-Nrf2 group (P < 0.01) (Figure 3A). In contrast, ΔΨm was significantly restored in the HG + CUR group compared to the HG group (*P* < 0.01) (Figure 3B). Additionally, the loss of ΔΨm was significantly reduced in the HG + CUR + shRNA-Nrf2 group compared with the HG + shRNA NC group (*P* < 0.01). These results suggest that CUR mitigates HG-induced mitochondrial ΔΨm loss in cardiomyocytes, thereby preventing further cell injury.

### 3.4 Effect of CUR on mPTP opening in HG-induced cardiomyocytes

**FIGURE 4:**
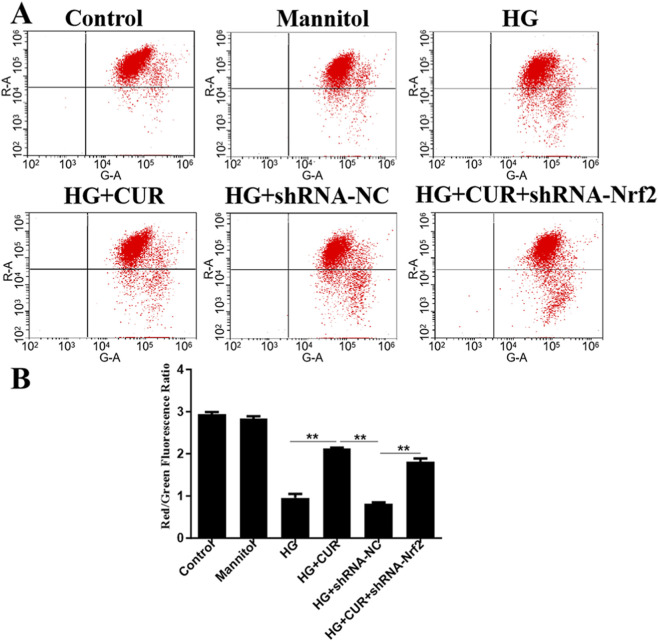
Effect of CUR on mPTP opening in cardiomyocytes induced by HG. **(A)** Western blot bands of cytc in cytoplasm and mitochondria across intervention groups. **(B)** Quantitative analysis of Western blot results (data are presented as mean ± S. E. M; The quantitative data were obtained from all three replicate experiments. ***P < 0.001, ΔΔ*P* < 0.01, ###*P* < 0.001 vs. Control group).

Western blot analysis was used to assess cytoplasmic and mitochondrial cytochrome c (cytc) protein expression, investigating the relationship between CUR-activated Nrf2 signaling and mitochondrial permeability transition pore (mPTP) opening (Figure 4A). In the control group, cytc levels were elevated in the mitochondria but minimal in the cytoplasm, indicating intact mPTP structure. In HG-induced cardiomyocytes, the mitochondrial-cytoplasmic cytc balance was disrupted, with increased cytoplasmic cytc and decreased mitochondrial cytc, particularly in the HG + shRNA NC group. Nrf2 activation partially restored cytc balance in the HG group (Figure 4B). This, along with the effects of Nrf2 on ΔΨm, suggests that Nrf2 signaling may partially inhibits mPTP opening under HG conditions.

### 3.5 Effect of CUR on cardiomyocyte apoptosis induced by HG

**FIGURE 5:**
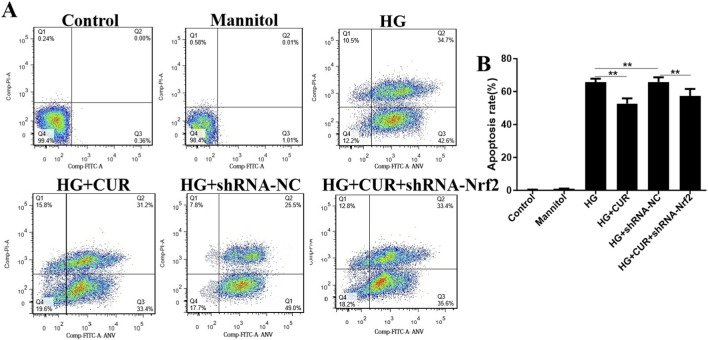
Analysis of HG-induced cardiomyocyte apoptosis by CUR. **(A)** Flow cytometry analysis of cardiomyocyte apoptosis across intervention groups. **(B)** Quantitative analysis of apoptosis across groups (data are presented as mean ± S. E. M; The quantitative data were obtained from all three replicate experiments. **P < 0.01).

Apoptosis was assessed by flow cytometry. Statistical analysis revealed no significant difference between the control and mannitol groups. Compared with the HG group, the apoptosis rate in the HG + CUR group decreased significantly (*P* < 0.01). Similarly, apoptosis in the HG + CUR + shRNA-Nrf2 group was significantly lower than in the HG + shRNA NC group (*P* < 0.01) (Figure 5B). These findings indicate that CUR reduces cardiomyocyte apoptosis via the Nrf2 signaling pathway.

### 3.6 Effect of Lv-shRNA-Nrf2 on cardiomyocytes

**FIGURE 6:**
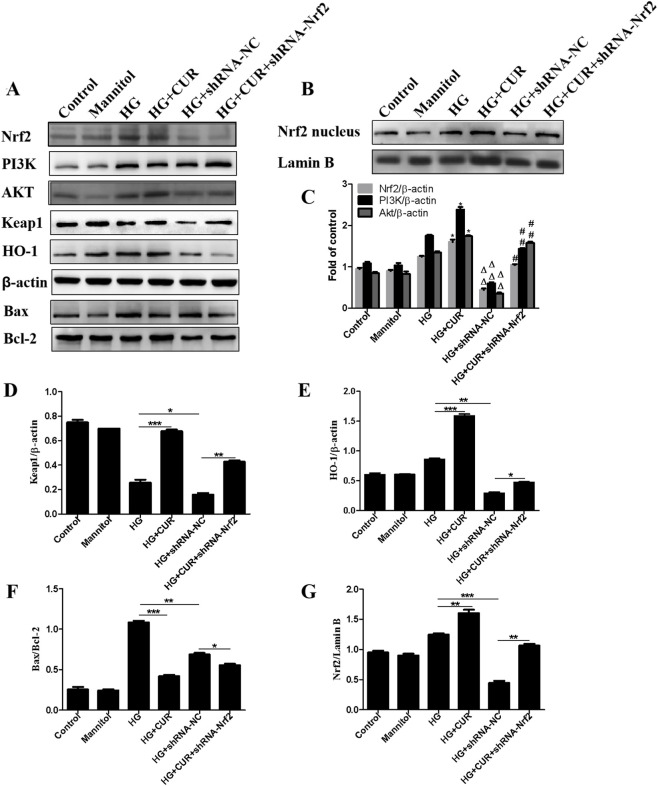
CUR activates the Nrf2 signaling pathway to protect against HG-induced myocardial injury. **(A)** Western blot bands of related proteins across intervention groups (Nrf2, Keap1, PI3K, AKT, HO-1, Bax, Bcl-2). **(B)** Western blot bands of nuclear Nrf2 across different groups. **(C–G)** Quantitative analysis of related proteins (Nrf2, PI3K, AKT, Keap1, HO-1, Bax, Bcl-2) (data are presented as mean ± S. E. M; The quantitative data were obtained from all three replicate experiments. *P < 0.05 vs. HG group; ΔΔ*P* < 0.01 vs. HG group; #*P* < 0.05, ##*P* < 0.01 vs. HG + CUR group).

Lv-shRNA-Nrf2 was used to infect primary rat cardiomyocytes. After 3 days, Western blot analysis revealed a significant reduction in Nrf2 protein expression in both the cytoplasm and nucleus, confirming the effective interference by Lv-shRNA-Nrf2 (*P* < 0.01) (Figures 6A–C,G). Statistical analysis showed that protein expression levels of PI3K, AKT, Keap1, HO-1, and Bcl-2 were significantly higher in the HG + CUR group compared to the HG group (P < 0.01) (Figures 6C–G). However, these protein levels were significantly reduced in the HG + shRNA NC group compared to the HG group. After HG + CUR + shRNA-Nrf2 treatment, this increase was suppressed (*P* < 0.01) (Figures 6C–G). Apoptosis in cardiomyocytes is tightly regulated by the balance between the anti-apoptotic protein Bcl-2 and the pro-apoptotic protein Bax. Under HG exposure, Bax levels increased and Bcl-2 levels decreased in cardiomyocytes. CUR treatment prevented the decrease in Bax and the increase in Bcl-2, mitigating the reduction in the Bcl-2/Bax ratio caused by HG (Figure 6F). These results suggest that CUR may inhibit cardiomyocyte oxidative stress and apoptosis by activating the Nrf2 signaling pathway.

## 4 Discussion

DCM is a severe complication of diabetes, primarily driven by hyperglycemia-induced oxidative stress, which leads to excessive ROS production, mitochondrial dysfunction, and subsequent cardiomyocyte apoptosis (Jia et al., 2018). In this study, we demonstrated that CUR effectively alleviates HG-induced myocardial injury through activation of the Nrf2 signaling pathway. This activation enhances antioxidant enzyme activities, preserves mitochondrial membrane potential (ΔΨm), and reduces ROS generation, collectively suggesting CUR’s cardioprotective potential under hyperglycemic conditions.

As shown in Figure 1, CUR significantly increased cell viability in HG-treated cardiomyocytes compared with untreated HG cells, with an optimal concentration observed at 40 μM. This finding is consistent with previous studies reporting cytoprotective effects of CUR in oxidative stress models, suggesting a specific dosage range in which CUR achieves maximum protective efficacy without cytotoxicity (Deng et al., 2025; Ulasov et al., 2022). The IC50 value of 38.17 μM at 36 h (Figure 1C) further supports this optimal dosage range, providing a basis for further investigation of CUR concentration-response relationships.

The effect of CUR on ROS production and antioxidant enzyme activity was confirmed in Figures 2A–E. CUR-treated cells exhibited lower ROS levels and higher activities of key antioxidant enzymes, such as HO-1, GSH-Px, and SOD, compared to untreated HG groups. This outcome aligns with the role of Nrf2 as a major transcription factor regulating antioxidant responses, crucial for counteracting oxidative damage (Hashemi et al., 2023; Yang et al., 2024). The upregulation of Nrf2 and its downstream targets by CUR in our study supports previous findings by Chen et al., which highlight Nrf2 as a critical regulator of cellular redox balance under diabetic conditions (Patel et al., 2020). These results align with other studies demonstrating CUR’s ability to activate Nrf2 and enhance cellular antioxidant defenses, further providing its therapeutic potential against DCM (Wei et al., 2023).

Mitochondrial integrity, as measured by ΔΨm, is another key indicator of cell health under oxidative stress. Figure 3 illustrates that CUR treatment stabilized ΔΨm in HG-exposed primary neonata rat cardiomyocytes, preventing mitochondrial depolarization. This stabilization effect suggests that CUR may mitigate HG-induced mitochondrial dysfunction, a primary contributor to DCM pathogenesis (Rahban et al., 2020). Our findings also revealed that CUR preserved mitochondrial integrity under HG conditions, as evidenced by the stabilization of ΔΨm (Figure 3) and the balanced distribution of cytochrome c between the cytoplasmic and mitochondrial fractions (Figure 4). These results suggest that CUR mitigates HG-induced mitochondrial outer membrane permeabilization, thereby reducing the release of pro-apoptotic factors. This effect is significant, as mPTP opening is a precursor to apoptotic signaling in damaged mitochondria, leading to cardiomyocyte apoptosis in diabetic hearts (Wei et al., 2022; Sarmiento-Ortega et al., 2025). These results corroborate studies by Zhang et al., who reported CUR’s potential to preserve mitochondrial function and prevent apoptotic pathways in oxidative stress models (Pan et al., 2025).

In addition to its antioxidant and mitochondrial effects, CUR treatment significantly increased the expression level of PI3K and AKT in HG-treated cardiomyocytes (Figure 6), indicating activation of the PI3K/AKT signaling pathway. This pro-survival pathway is known to cross-talk with Nrf2 signaling and may contribute to the cytoprotective effects of CUR under hyperglycemic conditions (Wu et al., 2022; Sadeghi et al., 2023). This combined activation is consistent with prior research indicating that CUR can activate PI3K/AKT in conjunction with Nrf2 to provide synergistic protective effects against oxidative damage (Xiang et al., 2020; Yu et al., 2016).

While our study provides valuable insights, several limitations should be noted. First, further studies using primary cardiomyocytes or animal models of DCM are necessary to confirm these findings. Additionally, although we identified key pathways such as Nrf2 and PI3K/AKT in CUR-mediated cardioprotection, the precise molecular mechanisms underlying their interaction remain incompletely understood. Although our results demonstrate that CUR modulates the expression of apoptosis-related proteins Bcl-2 and Bax (Figure 5), the precise molecular mechanisms linking Nrf2 and PI3K/AKT activation to the regulation of these Bcl-2 family members remain to be fully elucidated, given their pivotal roles in apoptosis regulation (Jin et al., 2022; Rajabi et al., 2024). Future studies employing targeted inhibitors or gene silencing approaches will be necessary to clarify the hierarchical relationships among these signaling pathways in CUR-mediated cardioprotection.

In conclusion, our study demonstrates that CUR exerts protective effects on HG-induced cardiomyocyte injury through activation of Nrf2 signaling, enhancement of antioxidant defenses, and preservation of mitochondrial function. The observed activation of the PI3K/AKT pathway further supports CUR’s cardioprotective role, suggesting a multi-faceted mechanism that could be harnessed for therapeutic interventions in DCM. Nevertheless, additional studies are needed to validate these effects *in vivo* and explore potential clinical applications for CUR in managing diabetic complications.

## Data Availability

The datasets presented in this study can be found in online repositories. The names of the repository/repositories and accession number(s) can be found in the article/supplementary material.
